# The Centrality of Trust in Academic Publishing Lies with the Corresponding Author

**DOI:** 10.5041/RMMJ.10525

**Published:** 2024-04-28

**Authors:** Jaime A. Teixeira da Silva

**Affiliations:** Independent Researcher, Kagawa-ken, Japan

**Keywords:** Corresponding author, inappropriate authorship, paper mills, sale of authorship, trust

**To the Editor:**

I have followed, with great interest, the passionate debate held between Lichtman,^1^ and Ashkenazi and Olsha^2^ in *Rambam Maimonides Medical Journal*. Lichtman put forward a curious and enlightening proposal to offer a fractional value to each author, depending on the value of their relative contribution, with the total amounting to 1, as a way to reduce authorship abuses, such as gift or guest authorship, which are two very prevalent forms of authorship abuses in academic publishing today.^3^

While Lichtman’s proposal is certainly a neat theoretical construct, one of the counter-arguments made by Ashkenazi and Olsha also has tremendous merit, worthy of some reflection, namely the potential for abuse. The latter two argued that the establishment of a power struggle within a team of authors may lead some individuals to pursue a higher relative score, independent of their actual contribution. One can easily imagine the potential for significant conflict that could arise between ambitious authors collaborating in multi-author teams or large-scale national or international projects. In such scenarios, several principal investigators or senior authors might vie for the top position on a scientific paper, each seeking to assert their leadership within the team. This power struggle, all for a mere fraction of a total of 1, results from an underlying culture of unhealthy reward systems imposed upon authors, which Ashkenazi and Olsha alluded to. Furthermore, if the total score for a paper is 1, and it is distributed between the authors according to Lichtman’s suggestion, the sole author of a paper would receive a 100% credit, achieving a score of 1. Conversely, a contributor in a 1000-author team, hypothetically receiving an equal share of the score to reflect an equal contribution (albeit an unrealistic option, but suggested here for simplicity), would receive a meager score of 0.001. Thus, if both parties (single author versus author who is part of a 1000-author team) were to be competitively observed, the latter author would have a 1/1000th fraction of the “contributor value” of the former.

Regardless how authorship contribution is weighed and assigned by and among members of a team, the corresponding author (CA) is ultimately responsible for transmitting information to a journal and its editors during the submission process, including data related to authorship. In addition to carrying a substantial moral and ethical obligation to the journal and publisher, the CA assumes a *de facto* leadership role by confirming the accuracy of authorship claims during the submission process.^4^ Hence, the decision of who should be assigned the role of CA should be carefully considered by the team of contributing authors. How then are co-CA positions determined, and how valid is this status if only a single person can submit a paper?^5^ If Lichtman’s relative weighting proposal were put into practice, would the CA receive a higher score than the other co-authors, based on the direct responsibilities associated with submission and communication with the journal?

In the real world, it is not uncommon to find that students with little or no career experience in research and publishing have been assigned the role of CA or co-CA on scientific papers. This choice carries a high potential for risk in the case of a crisis situation such as an ethics investigation: would the student CA mishandle the situation? Surprisingly, despite the centrality of the CA in the publication process, no remarks were directed to the role of the CA by Lichtman, or by Ashkenazi and Olsha, possibly because the focus of their letters lay elsewhere.

Independent of the truthfulness of the CA’s statements regarding authorship or the contribution of each author listed in an academic paper, the most significant vulnerability in academic publishing lies in the inability of editors and publishers to independently verify the truthfulness of CA-submitted statements, particularly regarding author contributions. Thus, fancily worded statements, highly granular contribution claims or scores (as would occur if Lichtman’s proposal were implemented for multi-author teams), complex, stringent, and inflexible submission requirements (e.g. mandatory ORCID) that merely waste the time and patience of a CA become functionally meaningless in a system that is unable to verify authorship contribution.^6^ Knowledge of these weaknesses by fraudulent authors would undoubtedly lead to abuses simply because fraud cannot be detected or caught. In extreme cases of authorship-for-sale schemes, as in paper mills,^7^ which erode the integrity of the publishing industry and trust in it, the CA merely serves the role of a tradesperson, liaising between clients (the paper mill and the journal, via the editor, guest editor, etc.).

In my opinion, unlike publishing yesteryear, the status of a CA in the age of paper mills may have become eroded to the level of being meaningless. If my perception is correct, since truthfulness lies in the hands of the CA, whose claims cannot be verified, trust in science may erode, so new solutions to authorship need to be devised.^8^

## References

[1] Lichtman MA (2023). Inappropriate journal authorship. Rambam Maimonides Med J.

[2] Ashkenazi A, Olsha O (2023). Inappropriate journal authorship, disputes, plagiarism, and mistrust in the institution: different beasts ... same problem. Rambam Maimonides Med J.

[3] Teixeira da Silva JA The abuse of authorship in the biomedical literature. Central Asian J Med Hypotheses Ethics 2023;.

[4] Teixeira da Silva JA, Dobránszki J, Van PT, Payne WA (2013). Corresponding authors: rules, responsibilities and risks. Asian Austral J Plant Sci Biotechnol.

[5] Teixeira da Silva JA Multiple co-first authors, co-corresponding authors and co-supervisors: a synthesis of shared authorship credit. Online Inf Rev 2021;.

[6] Teixeira da Silva JA (2023). How are authors’ contributions verified in the ICMJE model?. Plant Cell Rep.

[7] Pérez-Neri I, Pineda C, Sandoval H (2022). Threats to scholarly research integrity arising from paper mills: a rapid scoping review. Clin Rheumatol.

[8] Feine J, Jakubovics N (2021). Science in the spotlight: a crisis of confidence?. JDR Clin Trans Res.

